# Heteroduplex DNA Position Defines the Roles of the Sgs1, Srs2, and Mph1 Helicases in Promoting Distinct Recombination Outcomes

**DOI:** 10.1371/journal.pgen.1003340

**Published:** 2013-03-14

**Authors:** Katrina Mitchel, Kevin Lehner, Sue Jinks-Robertson

**Affiliations:** Department of Molecular Genetics and Microbiology, Duke University Medical Center, Durham, North Carolina, United States of America; Columbia University College of Physicians and Surgeons, United States of America

## Abstract

The contributions of the Sgs1, Mph1, and Srs2 DNA helicases during mitotic double-strand break (DSB) repair in yeast were investigated using a gap-repair assay. A diverged chromosomal substrate was used as a repair template for the gapped plasmid, allowing mismatch-containing heteroduplex DNA (hDNA) formed during recombination to be monitored. Overall DSB repair efficiencies and the proportions of crossovers (COs) versus noncrossovers (NCOs) were determined in wild-type and helicase-defective strains, allowing the efficiency of CO and NCO production in each background to be calculated. In addition, the products of individual NCO events were sequenced to determine the location of hDNA. Because hDNA position is expected to differ depending on whether a NCO is produced by synthesis-dependent-strand-annealing (SDSA) or through a Holliday junction (HJ)–containing intermediate, its position allows the underlying molecular mechanism to be inferred. Results demonstrate that each helicase reduces the proportion of CO recombinants, but that each does so in a fundamentally different way. Mph1 does not affect the overall efficiency of gap repair, and its loss alters the CO-NCO by promoting SDSA at the expense of HJ–containing intermediates. By contrast, Sgs1 and Srs2 are each required for efficient gap repair, strongly promoting NCO formation and having little effect on CO efficiency. hDNA analyses suggest that all three helicases promote SDSA, and that Sgs1 and Srs2 additionally dismantle HJ–containing intermediates. The hDNA data are consistent with the proposed role of Sgs1 in the dissolution of double HJs, and we propose that Srs2 dismantles nicked HJs.

## Author Summary

Chromosomal damage that occurs during normal cell division can be repaired using an intact sequence elsewhere in the genome as a template. This process, termed homologous recombination, is crucial for the repair of a particularly deleterious lesion, the DNA double-strand break. Although recombination is a repair process, it can also lead to exchanges of genetic material, generating crossovers (COs) between the involved chromosomes. Repair of the break without exchange of flanking DNA is called a noncrossover (NCO). As COs can uncover recessive mutations or result in large-scale genome rearrangements, understanding how the CO-NCO outcome is regulated is critical to issues of genome stability. The current study examines the distinctive mechanisms whereby three yeast DNA helicases—Mph1, Sgs1, and Srs2—contribute to the repair of a DNA double-strand break.

## Introduction

Faithful transmission of genetic information in mitotically dividing cells requires the repair of DNA damage that occurs from exogenous and endogenous sources. Damage to both strands of DNA can cause a double-strand break (DSB), as can replication of a damaged DNA template containing a nick. A single, unrepaired DSB can result in the loss of essential genes and lead to permanent cell-cycle arrest and cell death. To prevent these outcomes, DSBs are repaired by one of two pathways: error-prone nonhomologous end joining or error-free homologous recombination (HR). As the major DSB repair pathway in the yeast *Saccharomyces cerevisiae*, HR promotes high-fidelity repair through the use of an intact template DNA sequence. However, HR can also lead to loss of heterozygosity and gross chromosomal rearrangements and thus requires tight regulation.

**Figure 1:**
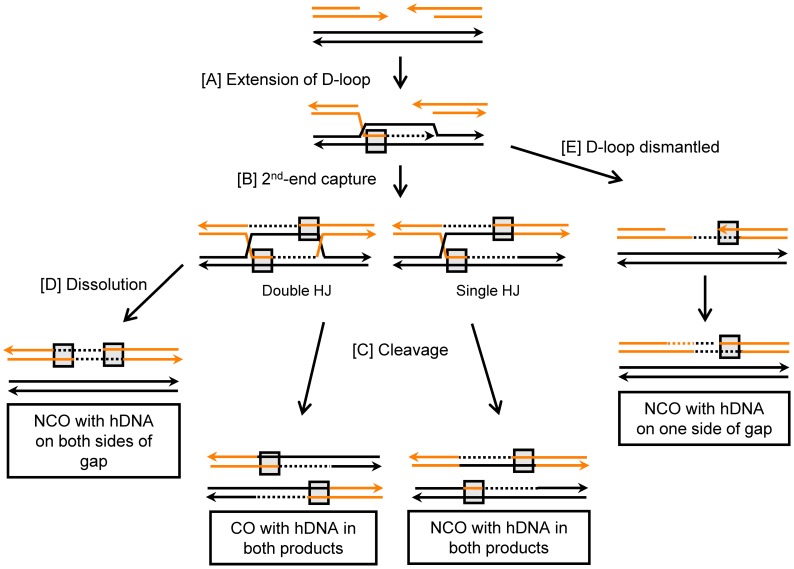
Gap-repair pathways. Single strands of DNA are represented by orange and black lines, and arrowheads indicate 3′ ends. Regions of hDNA are boxed, and newly synthesized DNA is depicted as dashed lines in the same color as the template allele. Additional detail is provided in the text.

To initiate HR, the 5′ ends of the DSB are resected to yield 3′ single-stranded regions of DNA (for reviews, see [1], [2], [3], [4]). These 3′ ends are coated with Rad51 to form nucleoprotein filaments that are competent to conduct a homology search and invade a donor duplex DNA molecule, promoting pairing with the complementary strand. Successful strand invasion of a homologous duplex results in the formation of a D-loop structure consisting of a region of heteroduplex DNA (hDNA) and a displaced single strand of DNA (Figure 1). New DNA synthesis occurs using the 3′ invading end as a primer, and this reaction enlarges the D-loop. Expansion of the D-loop, or its movement with the extending 3′ end [5], eventually exposes sequences complementary to the other side of the break (Figure 1A). In the canonical DSB repair (DSBR) model of recombination [6], annealing between the D-loop and the non-invading end of the DSB (“2^nd^ end capture”) results in the formation of a double Holliday junction (dHJ) intermediate (Figure 1B). Alternatively, if the annealed D-loop is nicked, an intermediate with a single HJ (sHJ) will be generated [4], [7]. In the gap-repair system used here, there is a strong dependence of CO events on the Rad1-Rad10 endonuclease [8], which would be consistent with D-loop nicking. HJ-containing intermediates can be resolved by cleavage (Figure 1C), and this process is generally assumed to yield either noncrossover (NCO) products that maintain the original linkages of DNA flanking the break, or crossover (CO) products in which the linkages of flanking DNA are switched. As an alternative to cleavage, a dHJ-containing intermediate can be “dissolved” to yield exclusively NCO products (Figure 1D) (reviewed in [9]). In lieu of engaging the second end of the DSB and subsequent HJ formation, the D-loop can be dismantled (Figure 1E). Annealing of the newly synthesized DNA to the non-invading 3′ end of the break then provides a template for the synthesis of the other strand of the damaged molecule. As this Synthesis-Dependent Strand-Annealing (SDSA) pathway does not go through an HJ-containing intermediate, it yields exclusively NCO products [10].

In *S. cerevisiae*, three 3′ to 5′ DNA helicases - Srs2, Sgs1 and Mph1 - have been implicated in regulating the outcome of mitotic DSB repair [11], and each increases the frequency of NCO events relative to CO events [12], [13]. Srs2 (*s*uppressor of *r*ad6 *s*ensitivity) was the first of the three helicases to be identified, and its gene was discovered in a screen for mutations that suppress the UV sensitivity of *rad6* strains [14]. Because suppression depends on the HR machinery, it was suggested that this helicase normally inhibits recombinational bypass of DNA lesions [15]. In spontaneous recombination assays, loss of Srs2 increases the rate of recombination, confirming that Srs2 can inhibit recombination [16], [17]. The anti-recombination activity of Srs2 has been attributed to its “strippase” activity, which removes the Rad51 protein from single-stranded DNA ends and thereby prevents strand invasion [18], [19]. However, when Srs2 function was examined in the context of an HO endonuclease-induced DSB, it was paradoxically found to play a pro-recombination role [20]. The loss of Srs2 not only decreased the overall level of DSB repair, it led to a proportional increase of COs among the recovered products [12], suggesting a specific role in NCO formation. Although it has been suggested that Srs2 directly dismantles D-loops to promote NCOs via SDSA [21], an alternative possibility is that its pro-recombination role reflects the removal of Rad51 from single-stranded DNA (ssDNA) ends, which would promote annealing between the 2^nd^ end of the DSB and the newly extended strand upon D-loop collapse. Consistent with this possibility, biochemical studies have shown that Rad51 complexed with ssDNA is a potent inhibitor of Rad52-mediated annealing reactions [22].

Sgs1 (slow growth suppressor) was identified based on genetic interactions with Top3, with *sgs1* mutations suppressing the genetic instability and slow growth of *top3* strains [23]. Sgs1 is a member of the RecQ family of 3′ to 5′ DNA helicases and is the ortholog of the human BLM helicase [23], [24]. Mutations in the *BLM* gene lead to the autosomal recessive disorder Bloom's syndrome, which is characterized by genetic instability and increased sister chromatid exchange [25]. Like Srs2, the Sgs1 helicase has multiple roles in recombination. First, Sgs1 acts with the endonuclease Dna2 to promote extensive 5′ to 3′ resection of the DSB ends (for a review, see [26]). Second, biochemical and *in vivo* studies suggest that Sgs1, together with Top3 and Rmi1, promotes NCO formation by dissolving dHJ-containing intermediates that could alternatively be cleaved to yield COs [27], [28]. Dissolution involves migration of the two HJs towards each other, followed by decatenation of the two linked strands. Consistent with a role in dHJ dissolution, loss of Sgs1 results in increased CO formation during repair of an HO-induced DSB [12].

*MPH1* was identified in a screen for mutants exhibiting a mutator phenotype [29], and the encoded protein is the ortholog of the human Fanconi Anemia protein FANCM [30], [31]. The participation of Mph1 in HR was initially inferred from epistasis analysis [32]. In its absence, the frequency of HO-induced COs was found to be elevated, but overall levels of repair were not affected [13]. The increase in COs was suggested to specifically reflect a loss of SDSA events, and consistent with this, Mph1 efficiently dismantles D-loops *in vitro* [33]. In a plasmid-based gap-repair assay, COs were similarly found to be elevated in the absence of Mph1, a function that may be partially dependent on the mismatch repair (MMR) complex MutSα [34].

Although biochemical studies have suggested specific roles for Srs2, Sgs1 and Mph1 in promoting either SDSA or dHJ dissolution, corresponding *in vivo* evidence has been lacking. In particular, prior studies have not been able to distinguish whether a given NCO product was produced by HJ cleavage, HJ dissolution or SDSA. To more rigorously assess the specific functions of the Mph1, Srs2 and Sgs1 helicases in NCO formation, gapped plasmids were transformed into wild-type and mutant strains that were defective for the MMR protein Mlh1 and contained a diverged chromosomal template for repair. We measured gap-repair efficiency, determined the CO-NCO distribution among repair events and sequenced both products of individual NCO repair events to detect regions of hDNA. As the location of hDNA can be used to infer the underlying molecular mechanism of NCO formation, the data provide novel insight into how recombination intermediates are processed by these helicases. These molecular analyses are consistent the presumed roles of these helicases and suggest additional functions as well.

## Results

**Figure 2:**
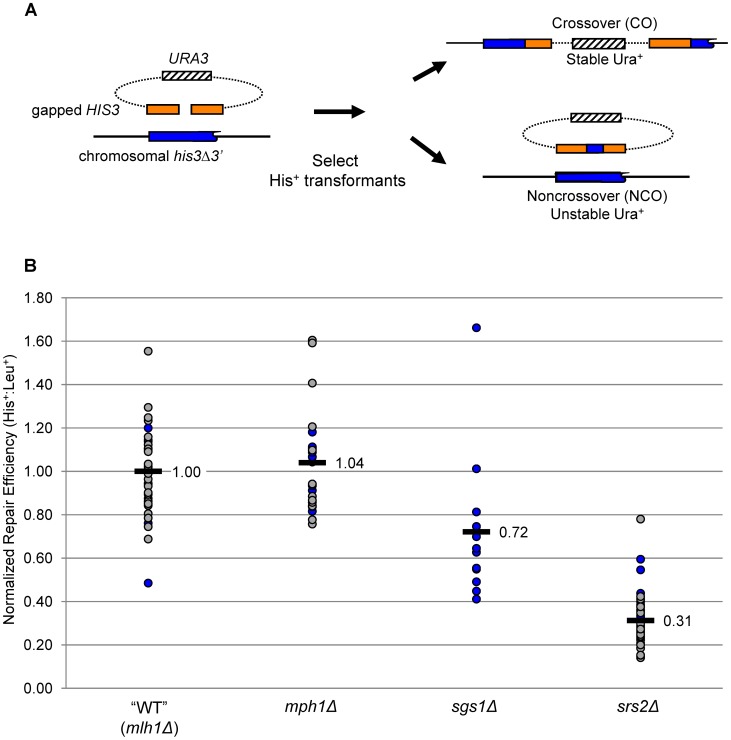
Gap-repair system and gap-repair efficiency in the absence of Mph1, Sgs1, or Srs2. A. Schematic of the gap-repair system, which detects both CO and NCO events. B. The efficiency of gap repair in WT and helicase-deficient strains, all of which are MMR-defective (*mlh1Δ*). Normalized ratios of His^+^ to Leu^+^ transformants in individual transformations are depicted by filled circles; ratios obtained with the first transformation mix are labeled in blue, and grey indicates ratios obtained with the second transformation mix. The mean is indicated with a black bar.

To analyze the roles of helicases in mitotic DSB repair, we used a transformation-based gap-repair system described previously (Figure 2A; [35]). Briefly, the introduced plasmid contained an 800 bp *HIS3* gene within which a centrally located, 8-bp gap was created by restriction digest. As a repair template, a *his3* allele missing the C-terminal 11 amino acids and containing 19 single nucleotide polymorphisms (SNPs) was inserted on chromosome V. The nearest SNPs are located 18 bp from each side of the gap. Because the donor sequence and plasmid are not identical, regions of hDNA formed during HR will contain mismatches. If not repaired, such mismatches segregate at the next round of replication, giving rise to a sectored colony with respect to the SNPs. To allow detection of hDNA, all experiments were conducted in an *mlh1*Δ, MMR-defective background. This background was chosen because our prior studies demonstrated that loss of Mlh1 affects neither the efficiency of gap repair nor the CO-NCO outcome [8]. The single-mutant, *mlh1*Δ parental strain thus served as the reference “wild-type” (WT), and all helicase-defective strains will be referred to hereafter by only their relevant helicase genotype.

The unique aspect of analyses done here is the tracking of hDNA in NCO products, as distinct patterns are predicted by SDSA, HJ dissolution and HJ cleavage. As illustrated in Figure 1, each of the NCO products generated by HJ cleavage is expected to contain a single region of hDNA. One region should be present on one side of the break in the repaired plasmid allele, and the other on the opposite side of the break in the chromosomal, donor allele. In contrast to NCO products generated by HJ cleavage, no change is expected to the donor duplex following HJ dissolution. Two regions of hDNA are predicted to be in the repaired allele, one on either side of the break. Finally, NCOs produced by SDSA are expected to contain a single region of hDNA in the repaired allele located specifically on the annealing side of the gap. As with HJ dissolution, no change to the donor allele is expected.

### Helicases and their effects on gap-repair efficiency

To control for variations in transformation efficiency, a linearized plasmid containing a *LEU2* marker was co-transformed with the gapped *HIS3* plasmid. His^+^ and Leu^+^ colonies were selected separately during each transformation, with the His^+^∶Leu^+^ ratio providing a measure of gap-repair efficiency. At least 12 independent transformations were done with the WT and each single-helicase mutant. To facilitate comparisons, the His^+^∶Leu^+^ ratio obtained in each individual transformation was divided by the mean His^+^∶Leu^+^ ratio obtained in the WT strain (see Materials and Methods). The normalized ratios are presented in Figure 2B, where the mean transformation efficiency of WT is 1.00. Relative to WT, the mean His^+^∶Leu^+^ ratio in the *mph1Δ* strain was 1.04, indicating that loss of Mph1 does not affect the overall efficiency of gap repair (p = 0.54 using a two-tailed Student's t-test). By contrast, the mean His^+^∶Leu^+^ ratio following transformation of the *sgs1*Δ strain was reduced 30% and that in the *srs2Δ* strain was reduced 3-fold relative to WT (p = 0.018 and p<0.0001, respectively). These data confirm that loss of either Sgs1 or Srs2 leads to decreased gap repair [8], and additionally demonstrate that loss of Mph1 has no effect in this system.

### Effects of individual helicase deficiencies on CO and NCO production

The gapped plasmid used in the transformation experiments contained an autonomously replicating sequence (*ARS*) but no centromere (*CEN*) sequence, allowing the repaired plasmid either to integrate into the chromosome with the repair template (a CO event) or to remain autonomous (a NCO event). These two outcomes were distinguished by examining the stability of the plasmid-encoded *URA3* marker, allowing His^+^ products to be partitioned into NCO and CO events (Figure 2A; [8]). Simply comparing the proportions of COs and NCOs in different genetic backgrounds (Figure S1) can be misleading, however, as it does not take into account changes in overall gap-repair efficiency. For example, an elevation in the proportion of COs could reflect either a specific gain in CO events with no effect on NCOs, a channeling of potential NCO products into the CO pathway or a specific loss of NCO products with no effect on COs. The efficiency of CO (or NCO) repair was thus calculated by multiplying the mean gap-repair efficiency by the proportion of CO (or NCO) events (Table 1). To allow statistical comparisons, the normalized His^+^∶Leu^+^ ratios in individual transformation experiments were multiplied by the proportion of COs or NCOs among gap-repair products, yielding a distribution of CO-type or NCO-type His^+^∶Leu^+^ ratios, respectively. The distributions in different strains were then compared using a two-tailed Student's t-test.

**Table 1:** Gap-repair efficiency and CO production in WT and helicase-deficient strains.

Relevant genotype	Plasmid repair efficiency^1^	CO events		NCO events	
		Proportion (%)	Efficiency^2^	Proportion (%)	Efficiency^2^
WT	1.00 (N = 36)	41/453 (9%)	0.09	412/453 (91%)	0.91
*mph1*Δ	1.04 (N = 24)	89/462 (19%)	0.20*	373/462 (81%)	0.84
*sgs1*Δ	0.72* (N = 12)	26/176 (15%)	0.11	150/176 (85%)	0.62*
*srs2*Δ	0.31* (N = 40)	86/343 (25%)	0.08*	257/343 (75%)	0.23*

All strains were MMR-defective (*mlh1*Δ). Asterisks indicate a significant difference when compared to WT using a Student's t-test (p<0.05).

1 Plasmid repair efficiency reflects the mean His^+^∶Leu^+^ ratio normalized to that obtained in the WT strain. N is number of independent transformations used to determine the mean.

2 Mean CO and NCO efficiencies were determined by multiplying the repair efficiency by the proportion of transformants that were categorized as CO and NCO events, respectively, based on plasmid stability.

In the WT yeast strain, only 9% of repair events were COs; the mean CO and NCO efficiencies were thus 0.09 and 0.91, respectively (Table 1). Although the overall efficiency of gap repair in the *mph1Δ* strain was indistinguishable from that in WT, the proportion of COs increased to 19%. The mean CO efficiency in the *mph1Δ* strain was thus 0.20, a change that was highly significant when compared to WT (p<0.0001). Even though there was a roughly compensatory decrease in proportion of NCOs in the *mlh1*Δ (from 0.91 in WT to 0.84), this did not translate into a significant change in mean NCO efficiency when compared to WT (p = 0.13). In the *sgs1*Δ strain, the proportion of COs among the repaired products increased to 15%. When the accompanying decrease in mean gap-repair efficiency in the *sgs1*Δ background was considered, however, there was no significant change in CO production relative to WT (0.09 and 0.11, respectively; p = 0.30). By contrast, the mean efficiency of NCOs decreased from 0.91 in the WT to 0.62 in the *sgs1*Δ strain, a change that was highly significant (p = 0.005). Finally, an even greater proportional increase in COs was observed in the *srs2Δ* strain: from 9% in WT to 25% of total repair events in the mutant. Taking into account the 3-fold decrease in overall gap-repair efficiency, however, the mean efficiency of CO formation was only very slightly reduced in the *srs2Δ* strain (from 0.09 to 0.08; p = 0.039). In contrast to the marginal effect on COs, the mean NCO efficiency decreased from 0.91 in WT to only 0.23 in the *srs2Δ* mutant (p<0.0001). Thus, with either an Sgs1 or Srs2 deficiency, the reduction in overall gap-repair efficiency reflects a strong reduction in NCO formation with little, if any, compensatory gain in COs. Upon loss of Mph1, however, there was a re-distribution of products types without a change in overall gap-repair efficiency.

### Differentiating NCO mechanisms based on the position of hDNA

**Figure 3:**
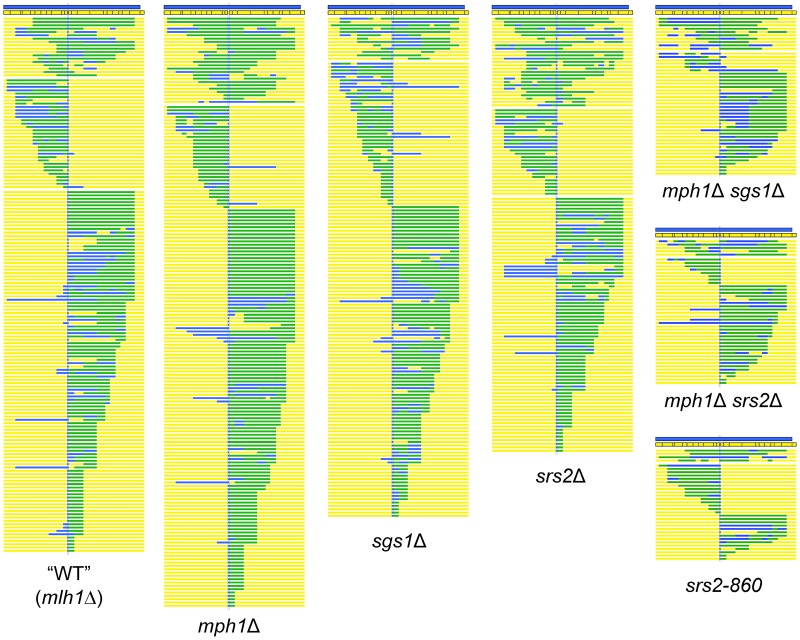
Position of hDNA in NCO products of WT and helicase-deficient strains. Each line represents the plasmid allele of a single NCO event; plasmid sequence is depicted in yellow, chromosomal sequence in blue and hDNA in green. The positions of the SNPs are indicated to scale within the chromosomal allele. Only those NCOs with hDNA detected on the plasmid allele are shown, as they were the samples used for statistical analysis. Samples are grouped by strain background (all strains were *mlh1Δ*) and arranged based on the position of hDNA.

Alterations in CO or NCO production could reflect an effect on the NCO-specific SDSA pathway, a change in the efficiency of forming HJ-containing intermediates and/or a change in how HJ-containing intermediates are resolved. To differentiate between these possibilities, a *HIS3*-containing *CEN* plasmid, which generates only viable NCO products, was used in transformation experiments. Both the plasmid and chromosomal alleles involved in individual gap-repair events were sequenced (Table S1). Of 249 NCO products sequenced from the WT strain, regions of hDNA were detected on the plasmid allele in 159 (Table 2). In 18 of these, hDNA was present on both sides of the gap (bidirectional hDNA), consistent with the hDNA pattern predicted by formation of an HJ-containing intermediate; the remaining 141 had hDNA on only one side of the gap (unidirectional hDNA), consistent with production via the SDSA mechanism (Figure 3). It should be noted the distribution of hDNA observed here when using the NCO-only plasmid is very similar to that previously reported using an *ARS*-containing plasmid [35], and confirms that ∼90% of NCOs are likely derived from SDSA. Although the corresponding chromosomal alleles were sequenced in each NCO event analyzed, none had the hDNA pattern predicted by HJ cleavage, confirming that HJ cleavage does not contribute significantly to NCO formation in this system (Table S1; [35]). To estimate the efficiencies with which HJ dissolution and SDSA occurred, the proportions of bidirectional and unidirectional hDNA tracts, respectively, among NCOs was multiplied by the mean efficiency of NCO production. In WT, the mean NCO efficiency of 0.91 was thus broken down into a bidirectional and unidirectional hDNA values of 0.10 and 0.81, respectively (Table 2).

**Table 2:** Efficiency of NCO events with unidirectional versus bidirectional hDNA in the repaired plasmid allele.

Relevant genotype	Plasmid repair efficiency	CO efficiency	NCO efficiency	Position of hDNA in repaired plasmid allele			
				Unidirectional		Bidirectional	
				Proportion (%)	Efficiency	Proportion (%)	Efficiency
WT	1.00	0.09	0.91	141/159 (89%)	0.81	18/159 (11%)	0.10
*mph1*Δ	1.04	0.20*	0.84	150/176 (85%)	0.71*	26/176 (15%)	0.12*
*sgs1*Δ	0.72*	0.11	0.62*	136/149 (91%)	0.56*	13/149 (9%)	0.05*
*mph1*Δ *sgs1*Δ	0.87* (N = 12)	0.19* (41/186)	0.67* (145/186)	36/47 (77%)	0.52*	10/47 (21%)	0.14*

All strains are *mlh1*Δ. Mean plasmid-repair, CO and NCO efficiencies were determined as in Table 1. The position of hDNA was determined by sequencing individual NCO events; proportions of unidirectional and bidirectional hDNA were derived using only those transformants in which hDNA was detected. One NCO event with the pattern of hDNA consistent with HJ cleavage was detected in the *mph1*Δ *sgs1*Δ background. Asterisks indicate a significant difference when compared to WT (p<0.05).

### Mph1 alters the distribution of uni- versus bidirectional hDNA among NCO products

The sequences of 242 NCO products isolated from the *mph1Δ* strain were analyzed and 176 hDNA tracts were detected. Of these tracts, 15% were bidirectional, and 85% were unidirectional (Figure 3; Table 2). When these proportions were multiplied by the mean NCO efficiency in the *mph1Δ* background, there was a small but significant increase in NCOs with bidirectional hDNA (from 0.10 in WT to 0.12 in *mph1Δ*; p = 0.002) and a corresponding reduction in NCOs with unidirectional hDNA (from 0.81 in WT to 0.71 in *mph1Δ*; p = 0.027). Thus, even though there was no significant decrease in total NCOs in the absence of Mph1, there was a shift from unidirectional to bidirectional NCO products.

### Sgs1 promotes NCOs with either unidirectional or bidirectional hDNA

In the absence of Sgs1, the overall gap-repair efficiency dropped to approximately 70% of the WT level, and this reflected a selective loss of NCO events with no compensatory gain in COs. The products of 285 NCOs isolated from the *sgs1*Δ strain were sequenced, 149 of which had detectable hDNA on the plasmid. Thirteen hDNA tracts were bidirectional, and 136 were unidirectional (Figure 3; Table 2). Taking into account the reduction in overall gap-repair efficiency in the *sgs1*Δ strain, NCOs with bidirectional hDNA decreased from 0.10 in the presence of Sgs1 to 0.05 in its absence (p<0.0001). NCOs with unidirectional hDNA also dropped significantly in the *sgs1*Δ strain (from 0.81 in WT to 0.56; p = 0.009). These data are consistent with the presumed role for Sgs1 in promoting NCO formation via dHJ dissolution and additionally indicate that Sgs1 promotes SDSA.

### Gap repair in the absence of both Sgs1 and Mph1

To examine the relationship between Sgs1 and Mph1 during gap repair, we constructed an *mph1*Δ *sgs1*Δ double-mutant strain. The mean gap-repair efficiency decreased significantly from ∼1.00 in the WT and *mph1Δ* strains to 0.87 in the double mutant (Table 2; p = 0.039 and p = 0.032, respectively), a decrease that was similar to that observed in the *sgs1*Δ single mutant (p = 0.21). The mean efficiency of NCO production in the *mph1*Δ *sgs1*Δ strain also was similar to that obtained in the *sgs1*Δ strain (p = 0.52) and significantly less that than in either the WT or *mph1Δ* background (p = 0.0001 and p = 0.012, respectively). Though the overall efficiencies of NCOs with unidirectional hDNA were similar in the *mph1*Δ *sgs1*Δ and *sgs1*Δ backgrounds (p = 0.6), the efficiency of the minority, bidirectional events in the double mutant was greatly elevated (p<0.0001), and only slightly different from that observed in the *mph1Δ* single mutant (0.14 and 0.12; respectively; p = 0.049). Altogether, the data suggest (1) that Sgs1 is more important than Mph1 for the production of NCO events with unidirectional hDNA and (2) that Sgs1 does not remove bidirectional hDNA-containing NCO intermediates that arise in the absence of Mph1. As will be elaborated further in the Discussion, we speculate that the elevated bidirectional hDNA in the *mph1Δ* background may reflect nicked HJ-containing intermediates, which are not expected to be substrates for the Sgs1-Top3-Rmi1 dissolvase.

### Srs2 promotes unidirectional and bidirectional hDNA classes of NCOs

In the absence of Srs2, the overall gap-repair efficiency decreased ∼3 fold and, as in the *sgs1*Δ strain, this reflected a specific reduction in NCO events (Table 1). Among 254 NCOs sequenced from the *srs2Δ* strain, hDNA was detected in 129. Bidirectional hDNA was present in 27 of these and unidirectional hDNA in the remaining 102 (Figure 3 and Table 3). As expected, the decrease in the efficiency of NCOs with unidirectional hDNA (from 0.81 in WT to 0.19 in *srs2Δ*; p<0.0001) was similar to the overall reduction in NCO events. There also, however, was a significant decrease in NCOs with bidirectional hDNA (from 0.10 in WT to 0.05 in the *srs2Δ* mutant; p<0.0001). To further explore the unexpected role of Srs2 in promoting the formation of bidirectional hDNA-containing NCO products, we examined gap repair in *mph1Δ srs2Δ* double- and *srs2-860* single-mutant backgrounds.

**Table 3:** Efficiency of NCO events with unidirectional versus bidirectional hDNA in the repaired plasmid allele.

Genotype	Plasmid repair efficiency (normalized His^+^∶Leu^+^)	CO efficiency	NCO efficiency	Type of NCO event			
				Unidirectional hDNA		Bidirectional hDNA	
				Proportion (%)	Efficiency	Proportion (%)	Efficiency
WT	1.00	0.09	0.91	141/159 (89%)	0.81	18/159 (11%)	0.10
*mph1*Δ	1.04	0.20*	0.84	150/176 (85%)	0.71*	26/176 (14%)	0.12*
*srs2*Δ	0.31*	0.08*	0.23*	102/129 (79%)	0.19*	27/129 (21%)	0.05*
*mph1*Δ *srs2*Δ	0.55* (N = 11)	0.16* (52/179)	0.39* (127/179)	38/43 (88%)	0.34*	5/43 (12%)	0.05*
*srs2-860*	0.80* (N = 12)	0.12* (26/178)	0.68* (152/178)	29/33 (88%)	0.60*	4/33 (12%)	0.08*

All strains are *mlh1*Δ. Mean plasmid-repair, CO and NCO efficiencies were determined as in Table 1. The position of hDNA was determined by sequencing individual NCO events; proportions of unidirectional and bidirectional hDNA were derived using only those transformants in which hDNA was detected. Asterisks indicate a significant difference when compared to WT (p<0.05).

### Gap repair in an *mph1Δ srs2Δ* double mutant

The mean gap-repair efficiency in the *mph1Δ srs2Δ* double mutant was 0.55, a value 2-fold less than that in the *mph1Δ* single (1.04; p<0.0001) but significantly greater than that obtained in the *srs2Δ* single mutant (0.31; p = 0.009). This suggests that in the absence of Mph1, the need for the pro-recombination activity of Srs2 is relaxed. A similar, intermediate value for mean NCO efficiency was observed in the double mutant (0.39) relative to the *mph1Δ* (0.84; p<0.0001) or *srs2Δ* (0.23; p = 0.015) single mutant. When NCOs obtained in double mutant were partitioned into those containing unidirectional or bidirectional hDNA (43 of 85 NCOs sequenced contained hDNA), the unidirectional class again had an intermediate value relative to the two single mutants. By contrast, the efficiency of producing the bidirectional hDNA class of NCOs in the double mutant was indistinguishable from that in the *srs2Δ* single mutant (p = 0.56) and significantly less than that in the *mph1Δ* single mutant (0.5 and 0.12, respectively; p<0.0001). Thus, in the absence of Mph1, Srs2 remains important for generating the bidirectional hDNA class of NCOs, while Sgs1 is dispensable (see above).

### Gap repair in an *srs2-860* mutant

The pro-recombination role of Srs2 in the gap-repair assay, which is specific for NCO events, could reflect its ability to unwind duplex DNA and/or its ability to remove Rad51 from nucleoprotein filaments; we will refer to these as its helicase and strippase activities, respectively. To examine the relevance of each activity to NCO production, we used the strippase-deficient *srs2-860* allele, which truncates the protein and eliminates the Rad51-interaction domain [36]. If only the helicase activity of Srs2 is important, then the efficiency of NCOs in the *srs2-860* strain is expected to be the same as in the WT background. If the strippase activity of Srs2 is relevant, however, then the efficiency of NCOs should be reduced in the *srs2-860* strain. The mean His^+^∶Leu^+^ ratio decreased 20% in the *srs2-860* strain relative to WT (p = 0.0051), but was nevertheless much greater than in the *srs2Δ* strain (0.80 and 0.31, respectively; p = 0.001). The *srs2-860* allele had a similar, intermediate effect on mean NCO production when compared to WT (0.39 and 0.91, respectively; p = 0.0006) or *srs2Δ* (0.39 and 0.23, respectively; p<0.0001). This intermediate effect extended to both the unidirectional (p = 0.004 and p<0.0001 when compared to WT and *srs2Δ*, respectively) and bidirectional hDNA classes of NCOs (p = 0.006 and p<0.0001 when compared to WT and *srs2Δ*, respectively). By contrast, the mean level of COs was elevated to 0.12 in the *srs2-860* strain, which was significantly higher than COs in either WT (0.09; p = 0.022) or *srs2Δ* (0.08; p<0.0001). This suggests that the strippase activity of Srs2 may promote NCOs at the expense of COs during gap repair.

## Discussion

**Figure 4:**
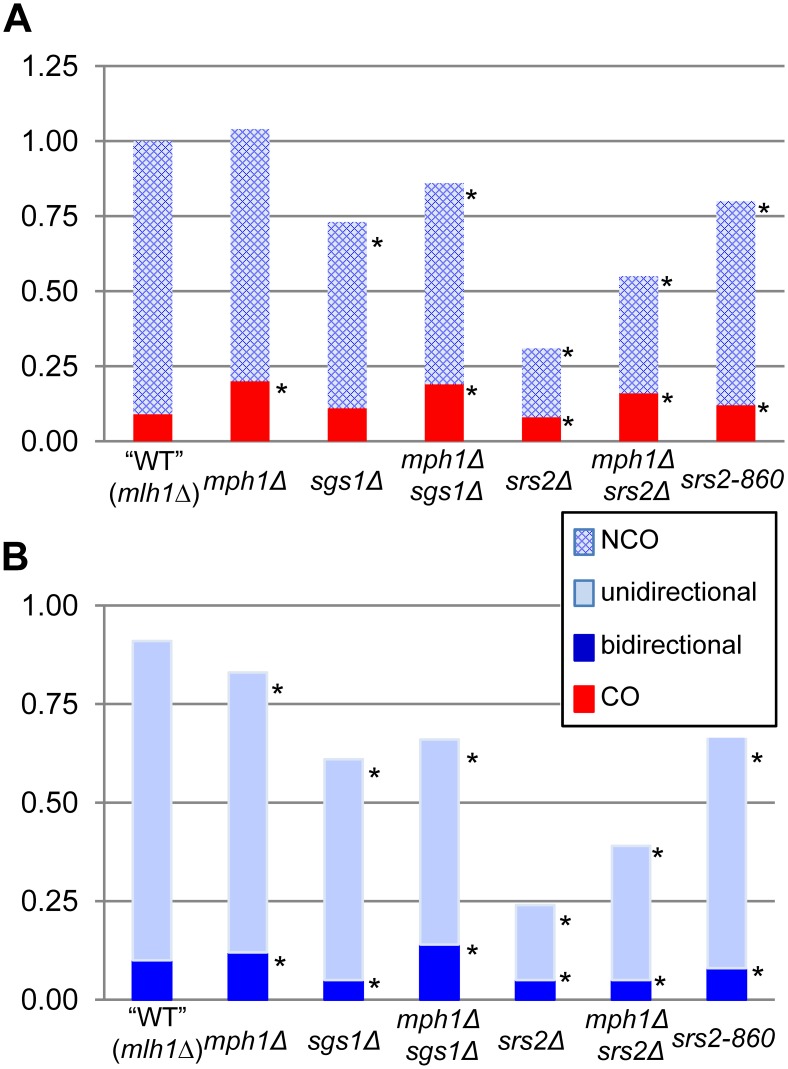
Distribution of gap-repair products in WT and helicase-deficient strains. Data presented in Table 1, Table 2, and Table 3 are summarized. A. The height of each bar indicates the normalized mean of each type of product. COs are in red and NCOs are in blue. Asterisks indicate p<0.05. B. NCOs with bidirectional hDNA are in dark blue, and NCOs with unidirectional hDNA are in light blue. Asterisks indicate p<0.05.

**Figure 5:**
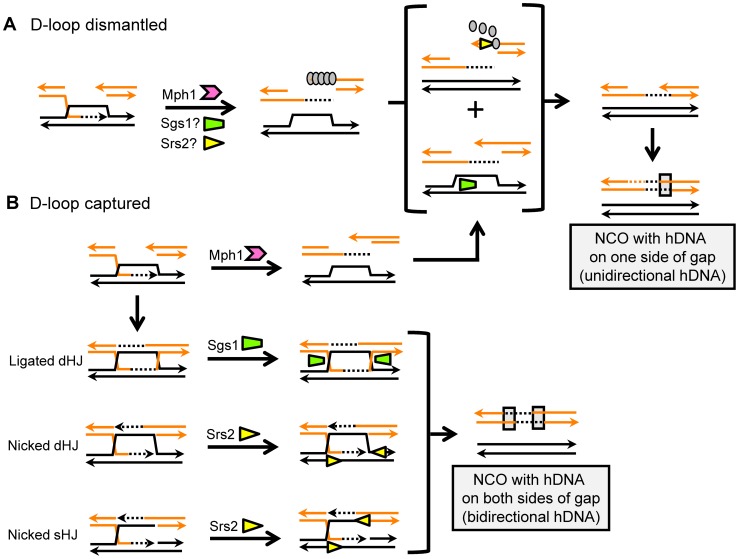
Models for helicase-mediated regulation of NCO formation. Sgs1, Srs2 and Mph1 are represented by green, yellow and pink symbols, respectively. A. Whereas all three helicases may be able to displace the invading end from the D-loop, Sgs1 and Srs2 are postulated to have additional activities during SDSA. Sgs1 may be required to rewind the invaded duplex, while the strippase activity of Srs2 may be important for 2^nd^-end annealing. Gray ovals represent Rad51, and its removal by Srs2 is indicated. B. If not dismantled by Mph1, the D-loop can be captured by the other end of the DSB and form an HJ-containing intermediate. A completely ligated dHJ is likely a substrate for Sgs1 complex, while a nicked sHJ or nicked dHJ may be a substrate for Srs2. The pattern of hDNA predicted by HJ cleavage is extremely rare in this system and does not contribute significantly to NCO events.

Most DSB repair studies have measured relative levels of CO and NCO products through physical analysis of large populations of repaired molecules [12], [13]. A distinguishing feature of the current study is the sequencing of both products of individual NCO events produced in a gap-repair assay, which allows inferences to be made about underlying molecular mechanisms and provides a unique tool for assessing specific structures acted on by candidate helicases. Specifically, the position of hDNA relative to the initiating gap indicates whether a given NCO event was generated by SDSA (“unidirectional” hDNA on only one side of the repaired gap), HJ cleavage (hDNA on one side of the gap in the repaired plasmid and on the other side of the gap in the chromosomal donor allele) or HJ dissolution (“bidirectional” hDNA on both sides of the gap in the repaired plasmid; see Figure 1). The data obtained in these analyses are summarized in Figure 4; panel A presents changes in the efficiencies of CO and NCO formation in various mutant backgrounds relative to WT, while panel B breaks down NCO events into those with unidirectional versus bidirectional hDNA. The major conclusions are as follows. First, as we reported previously [35], NCO production via HJ cleavage is extremely rare in a plasmid-based gap-repair assay; the hDNA pattern in ∼90% of events in WT was consistent with SDSA, and the remainder was consistent with HJ dissolution. Half of the bidirectional hDNA events were dependent on Sgs1 and the other half on Srs2, suggesting a difference in the “HJ” intermediate that is removed by each. Second, only Mph1 was dispensable for gap repair; in its absence, SDSA intermediates were diverted into an alternative pathway that yielded primarily CO events. By contrast, there was a decrease in overall gap-repair efficiency in either an *sgs1*Δ or *srs2Δ* background that primarily reflected loss of SDSA-derived NCOs. This suggests that there are distinct, pro-recombination roles for Sgs1 and Srs2 during SDSA. Third, loss of Sgs1 was associated with a reduction in bidirectional hDNA, providing novel molecular evidence for the presumptive role of this helicase in the dissolution of dHJs. Unexpectedly, there also was a decrease in efficiency of bidirectional hDNA products in an *srs2Δ* background. Finally, double-mutant analyses suggest that Mph1 acts prior to Srs2 during gap repair, as loss of Mph1 partially suppressed the gap-repair defect of an *srs2Δ* mutant. Below, we discuss the roles of Sgs1, Mph1 and Srs2 deduced here and incorporate these data into a model of how each helicase functions during NCO formation (Figure 5).

### Mph1 promotes SDSA at the expense of CO formation

Though an *mph1Δ*, *srs2Δ* or *sgs1*Δ mutant accumulates proportionally more COs during HO-induced DSB repair, an important distinction is that NCOs are diverted into COs only in an *mph1Δ* background [12]. The *mph1Δ* results reported here are consistent with the HO data, with the 2-fold increase in CO events being accompanied by a coordinate decrease in SDSA-type NCOs. While there are no data from higher eukaryotes that address CO-NCO distribution in the absence of Mph1, *Schizosaccharomyces pombe* mutants defective in the ortholog Fml1 similarly have normal efficiencies of gap repair accompanied by a strong proportional increase in COs [37]. These data place Mph1 at a key decision point during mitotic DSB repair, with its activity largely determining whether an intermediate has the potential to become a CO event. It indeed has been argued that Mph1 is the only one of the three helicases examined here that can unwind a mobile D-loop created by Rad51 [13], [38]. Though this suggests that Mph1 is the primary activity that dismantles D-loops in WT cells, NCO products with unidirectional hDNA are nevertheless produced efficiently in its absence. In the *mph1Δ* background, D-loop collapse could involve Srs2 and/or Sgs1, or some other helicase (Figure 5).

### Sgs1 is important for producing NCOs with unidirectional or bidirectional hDNA

As we reported previously using a similar gap-repair assay [8], there was a reduction in total gap-repair efficiency upon loss of Sgs1. The 2-fold increase in the proportion of COs observed in an *sgs1*Δ background reflected a specific loss of NCOs, with no corresponding gain in COs. The proportional gain in COs among recombination products is consistent with previous studies of yeast spontaneous and DSB-induced recombination [8], [12], [34], [39], as well as with studies in mammalian cells [40] and Drosophila [41].

In a time-course analysis of HO-induced DSB repair in yeast, the appearance of early NCOs, which were assumed to reflect SDSA, was not affected by loss of Sgs1, leading to the suggestion that Sgs1 specifically promotes a later dHJ dissolution pathway [12]. There have been numerous *in vitro* and *in vivo* studies that support a role for Sgs1, together with Top3 and Rmi1, in dHJ dissolution. *In vitro*, for example, human and Drosophila BLM-TopIIIα and yeast Sgs1-Top3 can dissolve dHJs [27], [42], [43]. *In vivo*, meiotic joint molecules formed in an *sgs1-ΔC795* mutant persist longer when cells are returned to mitotic growth, and their eventual resolution leads to proportionally more COs, consistent with dHJ dissolution by Sgs1 [44]. Furthermore, exposure of *sgs1* cells to DNA damage is associated with an accumulation of recombination-dependent X-shaped molecules [28], [45]. These molecules disappear when DNA is treated with bacterial HJ resolvases, suggesting that they correspond to fully-ligated HJs that are normally dissolved by the Sgs1-Top3-Rmi1 (STR) complex [28]. We have confirmed here that loss of Sgs1 is associated with a decrease in the specific class of NCOs predicted as the product of STR-driven dHJ dissolution: NCOs with bidirectional hDNA on the repaired molecule (Figure 5).

A reduction in the bidirectional hDNA pattern predicted by dHJ dissolution was expected upon loss of Sgs1, but a similar reduction in the unidirectional hDNA diagnostic of SDSA was not (Figure 5). Studies in Drosophila, however, have suggested a role for BLM in dismantling D-loops formed following P element excision [46], [47], and there is supporting biochemical evidence that human BLM binds and dismantles D-loops [48], [49]. Recent data suggest that the role of STR in D-loop disruption might also involve migration of the back end of the D-loop and/or rewinding of the invaded duplex [49], [50]. Either of these additional activities could explain why neither Mph1 nor Srs2 can fully substitute for Sgs1 during gap repair. We note that the reduction in unidirectional hDNA was no greater in the *mph1*Δ *sgs1*Δ double mutant than in the *sgs1*Δ single mutant, suggesting that Sgs1 and Mph1 work in the same pathway to promote SDSA. An interesting possibility is that, as the Mph1 helicase unwinds the invading strand, the catenating activity of STR is required to “rewind” the duplex that was part of the D-loop [50]. With regard to an SDSA-specific role for STR inferred here based on hDNA patterns, recent studies suggest that it is Sgs1 that promotes early SDSA-type NCOs in meiosis [51], [52].

### Roles of Srs2 in promoting NCO pathways during DSB repair

A pro-recombination role for Srs2 has been demonstrated in physical studies of HO-induced mitotic recombination [12], [20] and in a plasmid-based gap-repair assay similar to the one used here [8]. Furthermore, Srs2 loss has been associated with an increase in the proportion of COs produced during HO-induced DSB repair [12], during gap repair [8] and during spontaneous recombination [53]. Because only the early-appearing NCOs were lost following HO induction in an *srs2Δ* background, it was suggested that Srs2 promotes the NCO-specific SDSA [12]. In the current analyses, the efficiency of gap repair decreased 3-fold upon loss of Srs2, reflecting a specific loss of NCO events with no effect on CO events. Analyses of hDNA position in NCOs revealed a corresponding 4–5 fold reduction in SDSA-type products in the *srs2Δ* background, as well as a smaller, 2-fold decrease in bidirectional hDNA. These molecular data support that conclusion that SDSA is an early, Srs2-dependent pathway of DSB repair [12].

As inferred in HO assays [12], the pro-SDSA role of Srs2 in our gap-repair assay was much stronger than that of Sgs1. The most straightforward way for a helicase to promote SDSA is through the dismantling of an extended D-loop (Figure 5), but whether this is the most relevant function of Srs2 *in vivo* has been the subject of debate [13], [21]. The *srs2-860* allele used here encodes a protein that retains helicase activity but does not interact with Rad51, and hence is defective in the strippase activity that removes Rad51 from DNA [36]. A comparison of the overall gap-repair efficiencies in WT, *srs2Δ* and *srs2-860* strains suggests that the pro-recombination role of Srs2 likely reflects both activities. Importantly, the significant decrease in NCOs suggests that the disruption of Rad51 nucleoprotein filaments by Srs2 [18], [19] helps promote SDSA. This activity of Srs2 could be relevant for removing Rad51 from the 2^nd^ end of the resected break to allow the requisite Rad52-dependent annealing reaction and/or it could be necessary for D-loop disruption when Rad51 remains bound to duplex DNA within the D-loop (Figure 5). Either role would be consistent with the observation that overexpression of Rad51 in an *srs2Δ* background almost completely eliminates NCOs [12]. A late role for Srs2 in promoting 2^nd^-end annealing, however, seems more consistent with the observation that the pro-recombination for Srs2 is relaxed in the absence of Mph1 (Table 3). It should be noted that the 2^nd^-end engagement required to generate a dHJ could occur either through an annealing reaction or through a second, Rad51-catalyzed strand invasion event. The latter would be expected to be more efficient in an *srs2-860* background, which could explain the small increase in COs observed in this strain (Figure 4). Finally, the observation that neither Mph1 nor Sgs1 can substitute fully for Srs2 during DSB repair is consistent with a unique activity for this helicase.

The loss of the SDSA pattern of hDNA among NCO events in the *srs2Δ* background was expected based on prior studies, but the accompanying 2-fold reduction in the bidirectional hDNA pattern assumed to be diagnostic of dHJ dissolution was not. The unwinding of a 4-way structure that mimics an HJ by yeast Srs2 has been examined, and it was concluded that it is a no better substrate for yeast Srs2 than blunt-end duplex DNA [21]. A recent analysis of the putative homolog of Srs2 from *Arabidopsis thaliana*, however, reported that the helicase has significant activity against a nicked HJ [54]. We thus speculate that, in addition to a D-loop, a nicked sHJ or nicked dHJ is a cognate substrate for Srs2 *in vivo* (Figure 5). A nicked dHJ can be formed by a mechanism analogous to that assumed to occur when SDSA-mediated repair of a gapped plasmid requires that each end invade a template on a different chromosome [55], [56]. In the case of the assay used here, independent invasion of the *same* repair template by each end, followed by extension and unwinding - basically two independent SDSA reactions - would produce a repaired plasmid with bidirectional hDNA. A similar mechanism was previously invoked in a study examining the effect of homology length on the CO-NCO outcome during repair of HO-induced DSBs [57]. It should be noted that the contributions of Srs2 and Sgs1 to bidirectional hDNA among NCO products appear to be independent (each is required for ∼50% of these events in WT; Figure 4), which would be consistent with these helicases working on different structures: Srs2 on nicked single or double HJs, and Sgs1 only on fully ligated dHJs.

### Concluding remarks

Given the numerous roles that have been identified for the Mph1, Sgs1 and Srs2 helicases in basic DNA transactions, determining their specific activities once HR has initiated has been problematic. Although a gapped plasmid was used here to model DSB repair, it should be noted that both the efficiencies of repair and the distributions of repair products are completely consistent with those reported in HO-initiated chromosomal assays. It remains possible, however, that the roles of Mph1, Sgs1 and Srs2 inferred here may be specific to situations where the total homology between substrates is limited, a situation recently described for the Rad1-Rad10 endonuclease [58]. Through monitoring of hDNA among NCO products, the results presented here provide molecular confirmation that both Srs2 and Mph1 promote SDSA and that Sgs1 likely participates in the dissolution of dHJs. Importantly, additional roles for Sgs1 in promoting SDSA and for Srs2 in dismantling HJs have been inferred, broadening the potential range of activities of these helicases *in vivo*.

## Materials and Methods

### Media and growth conditions

Cells were grown nonselectively in YEPD (1% Bacto-yeast extract, 2% Bacto peptone, 2% dextrose) supplemented with 500 µg/mL adenine hemisulfate. Selective growth was on synthetic complete (SC) medium lacking the appropriate nutrient. Ura^−^ segregants were identified on SC plates containing 0.1% 5-fluoroorotic acid (5-FOA). All growth was at 30°C.

### Gap-repair experiments

A complete strain list is provided in Table S2. Helicase-defective derivatives of the haploid *mlh1*Δ strain SJR2157, which contains the diverged gap-repair template [8], were constructed by targeted gene deletion. The substrate for gap repair was generated by *Bss*HII linearization of either the *ARS*-containing plasmid pSR987 [35] or the *CEN/ARS*-containing plasmid pSR1015. pSR1015 was constructed by inserting an *Xho*I/*Xba*I *HIS3* fragment from pSR987 into *Xho*I/*Xba*I-digested pRS316 [59].

The OD_600_ of the exponentially growing cultures, each of which was derived from an independent colony, was measured to determine cell density. Six cultures with OD_600_ values between 0.7 and 1.0 were selected for parallel transformation using the protocol described previously [8]. Each experiment was repeated with at least six more cultures derived from independent colonies. His^+^ and Leu^+^ colonies were counted 5 days after selective plating. To avoid bias when partitioning recombinants into CO and NCO events, plates were divided into sections and every His^+^ transformant within a given section was picked. His^+^ transformants were frozen in 20% glycerol without prior purification, and an aliquot was grown nonselectively in YEPD prior to spotting an appropriate dilution on 5-FOA. Spots with full growth on 5-FOA after 3 days were scored as NCO events; those with no growth or only a few papillae were scored as CO events.

### DNA sequence analysis of recombinants

Transformations with pSR1015 and selection of His^+^ transformants were performed as described above. An aliquot of the frozen stock of each His^+^ colony was transferred to SC-his liquid medium and grown to saturation in 96-well microtiter plates. Following DNA extraction (http://jinks-robertsonlab.duhs.duke.edu/protocols/yeast_prep.html), the plasmid and chromosomal alleles were separately amplified with the appropriate primers (Table S3), and products were sequenced by the Duke Comprehensive Cancer Center DNA Analysis Facility. Sequence chromatograms were examined visually to detect the double peaks indicative of hDNA at a given SNP. Samples with only gene conversion or with no detectable sequence transfer were not included in further analysis because it was not possible to infer a recombination intermediate.

### Statistical analysis

Two different mixes of linear plasmids were used during the course of the transformation experiments reported here. The absolute His^+^∶Leu^+^ ratios obtained when using these mixes differed for the WT strain (mean ratios of 1.57 and 1.05), and depending on which mix was used for a specific mutant background, the individual transformation values were normalized to the corresponding WT mean. For the *mph1*Δ and *srs2*Δ strains, His^+^∶Leu^+^ ratios were generated with both mixes; for other mutant backgrounds, only a single mix was used in transformation experiments. For data analysis, normalized values of all individual transformations were pooled. The mean His^+^∶Leu^+^ ratios for the various events in different strain backgrounds were compared using a two-tailed Student's t-test (http://vassarstats.net/), and p<0.05 is considered significant.

## Supporting Information

Figure S1 Proportion of COs produced during gap repair in WT and helicase-deficient strains. The percentage of COs out of the total number of transformants is plotted by strain. The number of CO and NCO products were compared between WT and the helicase mutant strains, and a Fisher exact 2×2 probability test was used to calculate p-values.
(TIF)

Table S1 Sequence changes detected in the chromosomal allele of NCO gap-repair products.
(DOC)

Table S2 *Saccharomyces cerevisiae* strains employed in this study.
(DOC)

Table S3 Primer sequences for PCR amplification and sequencing of gap-repair products.
(DOC)
