# Supplementary figures and images for: Heteroduplex DNA Position Defines the Roles of the Sgs1, Srs2, and Mph1 Helicases in Promoting Distinct Recombination Outcomes
Source: PLoS Genet. 2013 Mar 14;9(3):e1003340. doi: 10.1371/journal.pgen.1003340 (PMC3597516; doi:10.1371/journal.pgen.1003340)

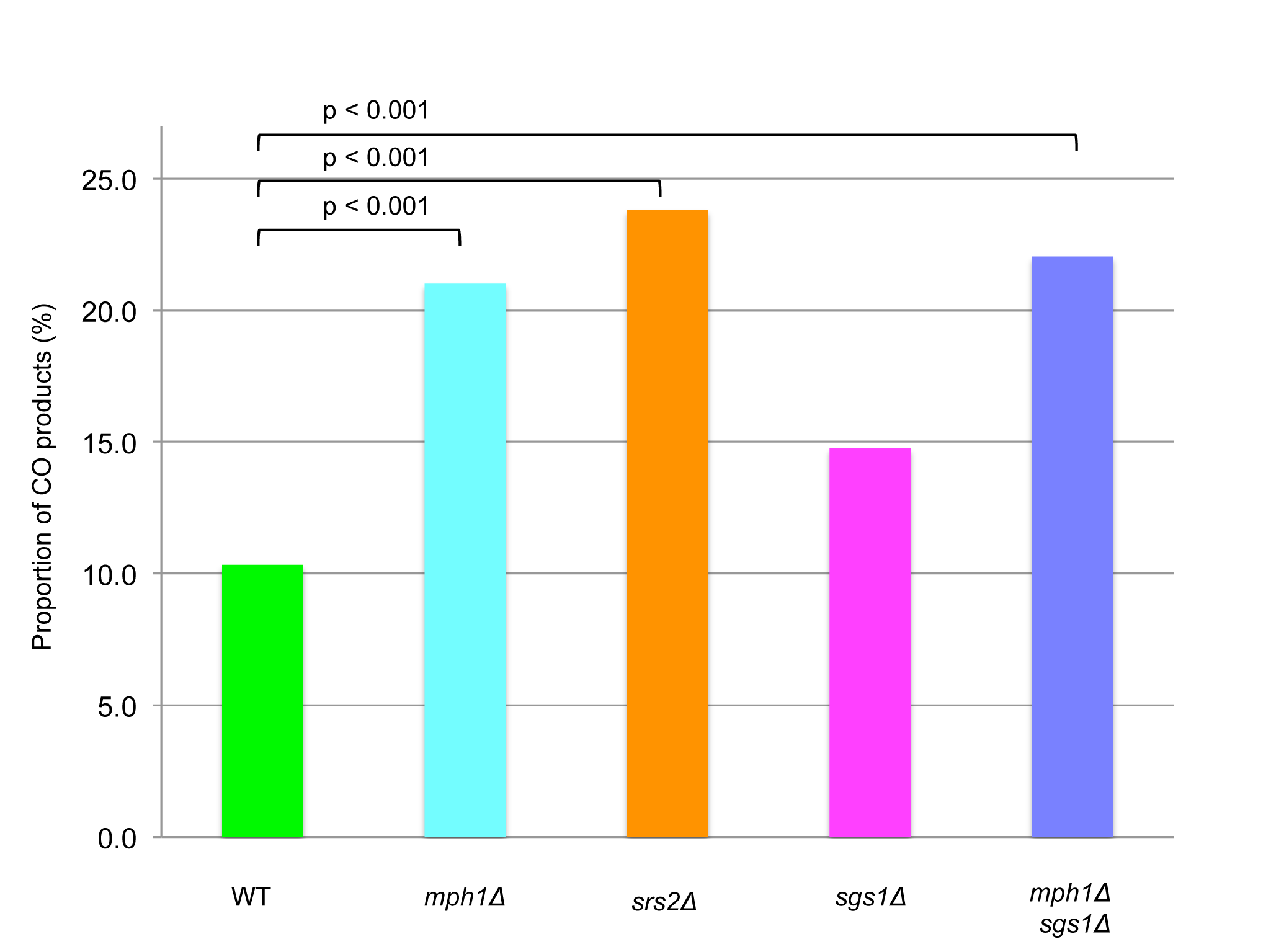

Supplement: Figure S1 — Proportion of COs produced during gap repair in WT and helicase-deficient strains. The percentage of COs out of the total number of transformants is plotted by strain. The number of CO and NCO products were compared between WT and the helicase mutant strains, and a Fisher exact 2×2 probability test was used to calculate p-values. (TIF) [file pgen.1003340.s001.tif]
