# Supplementary material for: Heteroduplex DNA Position Defines the Roles of the Sgs1, Srs2, and Mph1 Helicases in Promoting Distinct Recombination Outcomes
Source: PLoS Genet. 2013 Mar 14;9(3):e1003340. doi: 10.1371/journal.pgen.1003340 (PMC3597516; doi:10.1371/journal.pgen.1003340)
Supplement: Table S1 — Sequence changes detected in the chromosomal allele of NCO gap-repair products. (DOC) [file pgen.1003340.s002.doc]

**Table S1. Sequence changes detected in the chromosomal allele of NCO gap-repair products.**

| Genotype | Total number of events | Chromosomal sequence change | Corresponding plasmid sequence | Number of events |
| --- | --- | --- | --- | --- |
| Wild-type | 5 | Gene conversion | No plasmid sequence detected | 1 |
|  |  | hDNA | No plasmid sequence detected | 1 |
|  |  | hDNA | No hDNA detected (gap-only) | 1 |
|  |  | hDNA | Gene conversion | 1 |
|  |  | hDNA | Bidirectional hDNA | 1 |
| *mph1∆* | 5 | Gene conversion | Gene conversion | 1 |
|  |  | hDNA | No plasmid sequence detected | 2 |
|  |  | hDNA | Gene conversion | 2 |
| *srs2∆* | 9 | hDNA | No plasmid sequence detected | 5 |
|  |  | hDNA | No hDNA detected (gap-only) | 1 |
|  |  | hDNA | Gene conversion | 1 |
|  |  | Gene conversion | Gene conversion | 2 |
| *sgs1∆* | 7 | hDNA | No plasmid sequence detected | 3 |
|  |  | hDNA | hDNA at same location | 1 |
|  |  | Gene conversion | hDNA | 2 |
|  |  | hDNA | Bidirectional hDNA | 1 |
| *mph1∆ sgs1∆* | 8 | hDNA | Bidirectional hDNA | 1 |
|  |  | hDNA | No plasmid sequence detected | 4 |
|  |  | Gene conversion | Bidirectional hDNA | 1 |
|  |  | hDNA | hDNA at same location | 1 |
|  |  | hDNA | hDNA (consistent w HJ cleavage) | 1 |
| *mph1∆ srs2∆* | 3 | hDNA | No plasmid sequence detected | 2 |
|  |  | Gene conversion | No plasmid sequence detected | 1 |
| *srs2-860* | 6 | hDNA | Bidirectional hDNA | 2 |
|  |  | hDNA | hDNA at same location | 1 |
|  |  | hDNA | No plasmid sequence detected | 2 |
|  |  | Gene conversion | hDNA | 1 |
