# Supplementary material for: Heteroduplex DNA Position Defines the Roles of the Sgs1, Srs2, and Mph1 Helicases in Promoting Distinct Recombination Outcomes
Source: PLoS Genet. 2013 Mar 14;9(3):e1003340. doi: 10.1371/journal.pgen.1003340 (PMC3597516; doi:10.1371/journal.pgen.1003340)
Supplement: Table S2 — Saccharomyces cerevisiae strains employed in this study. (DOC) [file pgen.1003340.s003.doc]

**Table S2: *Saccharomyces cerevisiae* strains employed in this study.**

| Strain | Genotype | Plasmid used |
| --- | --- | --- |
| SJR2157 | *MATα ade2-101_oc_ his3∆200 ura3-Nhe lys2∆RV::hisG leu2-R can1::his3∆Kpn, 19 mut mlh1∆::kan* Gal^+^ | pSR987/pSR1015 |
| SJR2862 | *MATα ade2-101_oc_ his3∆200 ura3-Nhe lys2∆RV::hisG leu2-R can1::his3∆Kpn, 19 mut mlh1∆::kan sgs1∆::hyg* Gal^+^ | pSR987/pSR1015 |
| SJR3137 | *MATα ade2-101_oc_ his3∆200 ura3-Nhe lys2∆RV::hisG leu2-R can1::his3∆Kpn, 19 mut mlh1∆::kan mph1∆::hyg* Gal^+^ | pSR987/pSR1015 |
| SJR3208 | *MATα ade2-101_oc_ his3∆200 ura3-Nhe lys2∆RV::hisG leu2-R can1::his3∆Kpn, 19 mut mlh1∆::kan mph1∆::hyg sgs1∆::nat* Gal^+^ | pSR1015 |
| SJR3293 | *MATα ade2-101_oc_ his3∆200 ura3-Nhe lys2∆RV::hisG leu2-R can1::his3∆Kpn, 19 mut mlh1∆::kan mph1∆::hyg srs2∆::nat* Gal^+^ | pSR1015 |
| SJR3297 | *MATα ade2-101_oc_ his3∆200 ura3-Nhe lys2∆RV::hisG leu2-R can1::his3∆Kpn, 19 mut mlh1∆::kan srs2∆::hyg* Gal^+^ | pSR1015 |
| SJR3511 | *MATα ade2-101_oc_ his3∆200 ura3-Nhe lys2∆RV::hisG leu2-R can1::his3∆Kpn, 19 mut mlh1∆::kan srs2-860* Gal^+^ | pSR1015 |
