# Supplementary material for: Heteroduplex DNA Position Defines the Roles of the Sgs1, Srs2, and Mph1 Helicases in Promoting Distinct Recombination Outcomes
Source: PLoS Genet. 2013 Mar 14;9(3):e1003340. doi: 10.1371/journal.pgen.1003340 (PMC3597516; doi:10.1371/journal.pgen.1003340)
Supplement: Table S3 — Primer sequences for PCR amplification and sequencing of gap-repair products. (DOC) [file pgen.1003340.s004.doc]

**Table S3. Primer sequences for PCR amplification and sequencing of gap-repair products.**

| Primer | Sequence | Description |
| --- | --- | --- |
| M45 (T3) | 5’- CGC CAA GCT CGG AAT TAA C 3’ | Amplification of repaired *HIS3*; anneals to flanking vector sequence |
| M46 (T7) | 5’- GTA ATA CGA CTC ACT ATA GGG CG 3’ | Amplification of repaired *HIS3*; reverse sequencing primer; anneals to flanking vector sequence |
| B32 (Can1-388) | 5’- CGC CGA CAT AGA GGA GAA G 3’ | Amplification of *his3∆3’*; anneals to flanking *CAN1* sequence |
| M43 (CanR) | 5’- CTA ATC CAT GCC GCC AGT GGA AC 3’ | Amplification of *his3∆3’*; anneals to flanking *CAN1* sequence |
| R60 (His3R+443) | 5’- TGC AAA CCA AGT TCG ACA AC 3’ | Reverse sequencing primer; anneals to *HIS3* sequence |
